# Influence of Molecular Structure of POM on Processability Within Metal Injection Molding

**DOI:** 10.3390/polym17192621

**Published:** 2025-09-28

**Authors:** Thomas Forstner, Simon Cholewa, Tobias Früh, Dietmar Drummer

**Affiliations:** 1 Institute of Polymer Technology, Friedrich-Alexander-Universität Erlangen-Nürnberg (FAU), Am Weichselgarten 10, 91058 Erlangen, Germany; 2 Oechsler AG, Matthias-Oechsler-Straße 9, 91522 Ansbach, Germany

**Keywords:** metal injection molding, feedstock, polyoxymethylene, homopolymer, copolymer, melt viscosity, processing stability

## Abstract

Metal Injection Molding (MIM) is based on the processing of highly filled polymers via the well established polymer injection molding process. It offers a highly efficient processing route for the indirect manufacturing of especially small and complex metal parts. In this regard, polyoxymethylene (POM) is often used as a primary binder component in MIM feedstocks due to its high debinding rate through a time-saving catalytic debinding process, utilizing the acid-catalyzed degradation of POM for polymer removal. However, thermally induced degradation of POM under processing conditions can also lead to changes in processing behavior, which is particularly important in highly filled polymers due to their already challenging processability. In this context, the present work demonstrates the impact of POM homopolymers (POM-H) and copolymers (POM-C) with varying viscosities on feedstock characteristics, their influence on the thermal processing stability, and their significance for the properties of the green parts. Within the study, the thermal degradation of both material types was assessed by viscosity measurements and thermogravimetry, with POM-H exhibiting more significant degradation compared to the thermally more stable POM-C, especially at higher temperatures. Catalytic debinding performance was found to be adequate for all materials. However, lower viscosity POM-C grades are preferred to optimize processability in MIM.

## 1. Introduction

**Figure 1:**
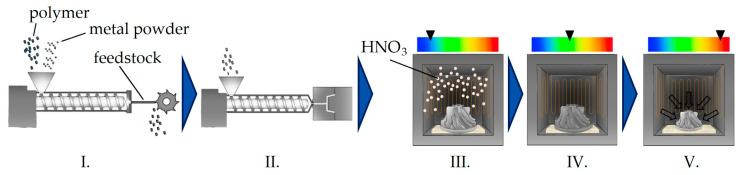
Processing scheme for a typical MIM process, involving catalytic debinding: (**I**). compounding of the feedstock, (**II**). injection molding, (**III**). catalytic debinding, (**IV**). thermal debinding; (**V**). sintering.

The indirect manufacturing of metal or ceramic parts by means of polymer processing offers great potential for an efficient, near-net-shape production of small and complex parts for numerous applications within mechanically or chemically challenging situations, as well as in high temperature environments. The possibilities for creating sintered metal or ceramic parts through polymer processing technologies are various and are available for a broad range of materials [1]. In this regard, indirect additive manufacturing via filament-based material extrusion (MEX) [2] especially addresses small quantities with a simultaneously high degree of geometrical complexity, for example, within rapid prototyping and rapid tooling [3]. Powder injection molding (PIM) [4,5] and powder extrusion molding (PEM) [6], on the other hand, are primarily focused on high production volumes for complex-shaped medical tools, automotive parts, and consumer articles in the case of PIM [5] and on two-dimensional products such as tubular profiles [7] and electrodes [8] in PEM, for example. Metal parts fabricated by metal injection molding (MIM) come with high densities and comparable mechanical properties in relation to conventionally manufactured parts [9] and can also be heat treated to further modify their specific properties to enhance ductility or hardness [9]. All of these manufacturing techniques, however, rely on the processing of highly filled polymers and follow the same process pattern for the indirect manufacturing of metals or ceramics. After the feedstock compounding and an original shaping step, this scheme usually involves a single- or multi-step debinding, which removes the polymer fraction from the part, followed by a sintering step [4] (pp. 1–23) to obtain the final part. The general processing scheme for MIM, based on a catalytically debindable binder system, is depicted in Figure 1.

The processing characteristics of MIM feedstocks throughout the whole process chain are determined by their binder composition [10] as well as by the particle characteristics of the sinterable filler in terms of size [11,12,13], shape [12,14], and quantity [15]. In general, the feedstock should have a sufficiently low viscosity to allow easy form filling [14] and a uniform distribution of the particle fillers resulting from a sufficient inter-particle spacing [16]. To match those needs, the binder is usually composed of multiple components and contains processing aids like waxes [17] or surfactants like stearic acid [18] in addition to the main component in most cases to precisely tailor the processing behavior. The choice of the main binder component, however, also defines the debinding process and, therefore, significantly affects the whole process chain. One of the most popular main binder materials in MIM is polyoxymethylene (POM), which is industrially used in the Catamold^®^ system by BASF and is also addressed in multiple research works [10,19,20]. POM is suitable for the catalytic debinding process, which enables significant time-saving in comparison to other debinding strategies like solvent or pure thermal debinding [21] and also allows efficient debinding of thick-walled parts [18]. In catalytic debinding, the polyacetal chain is broken down into formaldehyde by acid-catalyzed degradation through an ether cleavage in a nitrogen atmosphere enriched with nitric acid [22] or oxalic acid [23] vapor. Even though the decomposition behavior of POM, which is given by its molecular structure, is beneficial for the primary debinding of MIM feedstocks, lacking thermal stability as a result of depolymerization and autoxidation processes within the processing temperature range of POM also arises from its chain chemistry [24,25]. These processes are dependent on the molecular weight [24] of the polymer. Furthermore, POM-homopolymers (POM-H) are known to be thermally less stable than their copolymeric counterpart (POM-C) [26,27], even though both types are usually terminally stabilized to improve thermal stability [25,26,28,29]. Nonetheless, both types are prone to thermal-oxidative induced degradation as already mentioned, which may influence processing behavior due to unintentional degradation resulting from thermal exposition and mechanical stress during processing at an early stage depending on the POM type used. This affects the molecular weight distribution [27] and may lead to an altered rheological behavior of the polymer, which ultimately could cause problems in the already challenging MIM process.

Although the degradation behavior for neat POM-H and POM-C is already a well studied topic, there are no systematic investigations regarding the significance of the POM type in the field of MIM yet. In this context, existing works involving POM either focus on one POM type [30,31] or do not further specify the used POM in detail in the binder composition [19]. However, this is a crucial factor to know in order to enhance processability and to ensure sufficient reproducibility of injection-molded green parts. Therefore, the present study aims to create a generally better understanding of interactions of different molecular structures of POM in the form of the POM type and their degradation behavior within the context of MIM and their impact on processability.

## 2. Experimental

### 2.1. Materials

**Figure 2:**
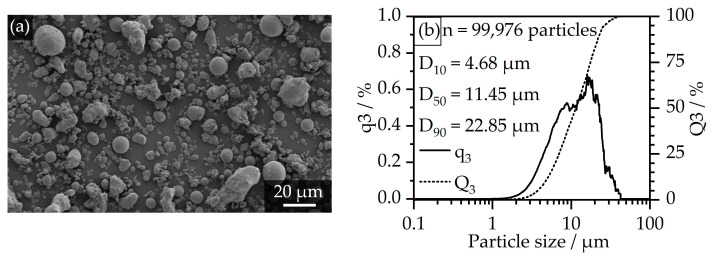
Particle characteristics of the 1.6523 case-hardening steel powder: (**a**) SEM image of the particles at 500× magnification; (**b**) volumetric particle size distribution determined on a Morphologi G3 by Malvern Panalytical (Malvern, United Kingdom) at 20× magnification.

For this study, a multi-component backbone-binder system for catalytic debinding was employed for the metal feedstocks. All feedstocks are composed of polyoxymethylene, high-density polyethylene (PE-HD), and stearic acid (SA) at a constant volumetric ratio between the different components within the binder of 79/16/5 (POM/PE-HD/SA). As the main binder component, different POM types were used in order to investigate their significance on feedstock and processing properties in MIM. In this regard, two POM-homopolymers and two POM-copolymers with comparable viscosities between the two classes were employed. In the following, the feedstocks, based on the lower viscosity POMs, will be declared as “POM-H/C low” while the higher viscosity POMs will be declared as “POM-H/C high”. As a backbone, which remains present after the catalytic debinding step, a commercially available PE-HD with an MFR of 8 g/10 min at 190 °C and 2.16 kg was employed. Stearic acid, which is often used for feedstocks in PIM [11] or MEX [32] amongst other processing aids like paraffin [12] and other waxes [18,33], was used as an additive. As a sinterable component, 1.6523 steel powder was employed. The filler content within the feedstock matched a constant amount of 60 vol.-%. The particle size distribution characteristics are typical of powders used in MIM [13] and feature a D_10_ = 4.68 µm, a D_50_ = 11.45 µm, and a D_90_ = 22.85 µm. The metal filler can be described as mostly spherical with a fraction of irregularly shaped and agglomerated particles. The particle characteristics are depicted in Figure 2.

### 2.2. Processing

The materials were compounded to obtain four different metal feedstocks for further characterization and for processing in MIM. Before compounding, the POM components were dried at 70 °C for 12 h and subsequently dry blended with the other binder components using a tumbling mixer. For filler incorporation, a co-rotating twin screw extruder ZSE HP 27 from Leistritz (Nuremberg, Germany) was used with temperatures of 160–200 °C from hopper to die. The strands obtained from compounding were cooled down on a cooling plate and pelletized for further processing.

Metal injection molding was carried out on an Allrounder 370 U by Arburg GmbH & Co. KG (Loßburg, Germany), which was equipped with an 18 mm diameter screw on the horizontal injecting unit. For the evaluation of feedstock processability, plate specimens with a length and width of 50 mm and a thickness of 2 mm were manufactured using a film gate. The parameters for injection molding were determined in preliminary tests and can be taken from Table 1.

For catalytic debinding, the samples were placed in an EBO catalytic debinding furnace by Carbolite Gero (Neuhausen, Germany) at 110 °C at a flow of 1400 L/h of nitrogen with the addition of 75 mL/h nitric acid within the debinding stage. Debinding times were varied from 3 min, 5 min, 10 min, 20 min, 40 min to 80 min to evaluate the progress.

### 2.3. Methods

The thermal stability of the POM materials and the debinding behavior of the compounded feedstocks were evaluated via thermogravimetric analysis (TGA). The measurements were conducted using a TGA Q5000 from TA-Instruments (New Castle, NSW, Australia). The samples weighed approximately 20 mg originating from the neat POM granulates for the evaluation of thermal stability and 60 mg originating from the compounded and pelletized feedstock granules for the evaluation of thermal debinding behavior. The procedures for the determination of thermal degradation comprised dynamic and isothermal experiments and were carried out under a constant flow of artificial air. Within the dynamic measurement, the material was heated up at a rate of 10 K/min to a maximum temperature of 650 °C. The isothermal protocol comprised a heating phase at a rate of 10 K/min up to the desired temperatures of 200 °C or 220 °C and an isothermal step of 60 min at these temperatures. Thermal debinding behavior was assessed through a dynamic measurement at 10 K/min up to a maximum temperature of 650 °C while constantly purging with nitrogen to simulate process conditions.

Rotational rheometry was employed for material characterization of the neat POM materials and for observing the thermally induced structural changes within the melt state of the materials. All measurements were conducted in oscillatory mode using a parallel plate setup with plates measuring 25 mm in diameter and a constant gap between the plates of 1 mm on a Discovery HR-2 rheometer by TA-instruments (New Castle, NSW, Australia). The linear viscoelastic region was determined for all materials through strain sweep experiments prior to all measurements. As a result, all measurements were performed within the linear viscoelastic region at 1% strain. For general material characterization, frequency sweeps were performed at temperatures of 180 °C and 200 °C for frequencies between 0.1 and 628 rad/s. Degradation behavior was evaluated via time-sweep measurements at a constant frequency of 10 rad/s and temperatures of 200 °C and 220 °C over a period of 60 min.

The particle–matrix adhesion was assessed in scanning electron microscopy (SEM) using a Gemini Ultra-Plus by Carl Zeiss (Oberkochen, Germany). The compounded feedstock strands were broken, and the exposed breaking surfaces were sputtered with gold. They were observed using a secondary electron detector at magnifications of 400× and 2000× at an acceleration voltage of 10 kV.

The catalytic debinding was evaluated by differential weighing of the granular feedstock samples before and after the process. Three samples were debound for each debinding time, which weighed around 40 g each.

## 3. Results and Discussion

### 3.1. Rheological Characterization

**Figure 3:**
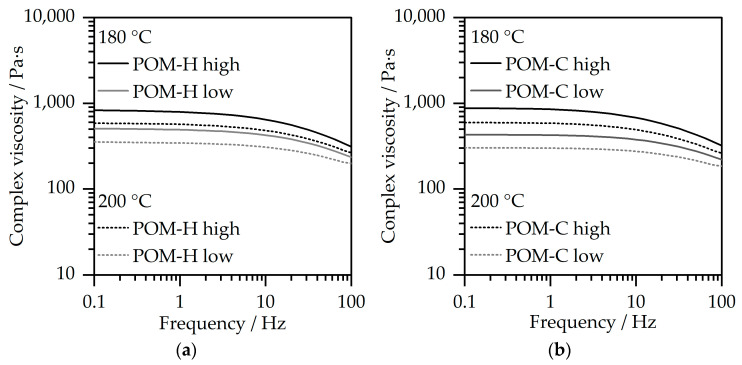
Frequency-dependent complex viscosity of neat POM-materials at 1% strain in nitrogen for 180 °C and 200 °C for (**a**) POM-H and (**b**) POM-C with high and low viscosity.

The rheological characterization was conducted for an evaluation of the shear-rate-dependent viscosity of both POM types for high and low viscosity within the processing-temperature range, and is depicted in Figure 3a for POM-H and in Figure 3b for POM-C.

The measurements show a similar shear-thinning behavior for all materials under investigation, which is a typical behavior for polymeric materials. Furthermore, the complex viscosity decreased with rising temperature for all materials. This behavior was more pronounced for the higher viscosity materials. In general, the shear-rate-dependent complex viscosities for POM-H as well as for POM-C can be described as comparable within the examined area, which is important for the further comparability within the processing of the highly filled compounds. This allows for an evaluation of the processing based on the general structure of the POM for the two general groups of viscosities.

### 3.2. Thermal Stability

The thermal stability was evaluated by TGA and additionally by time-sweep experiments in rotational viscosimetry to further describe the degradation behavior of POM.

#### 3.2.1. Thermogravimetrical Analysis (TGA)

**Figure 4:**
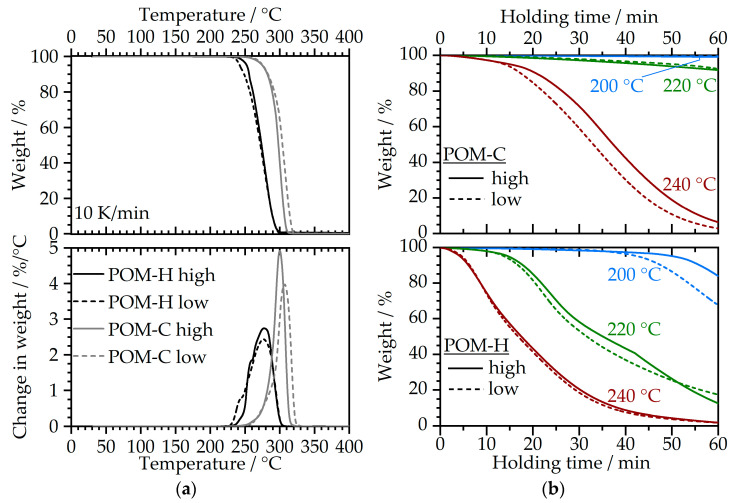
Temperature- and time-dependent decomposition behavior of neat POM materials in air atmosphere for (**a**) a dynamic heating at 10 K/min and (**b**) isothermal holding for 60 min at 200 °C, 220 °C and 240 °C after a heat up at 10 K/min.

Dynamic TGA measurements are shown in Figure 4a, while Figure 4b demonstrates the isothermal degradation behavior for different temperatures.

Under dynamic heating conditions, POM-H materials exhibited an early onset of thermal degradation, with decomposition starting at approximately 230 °C. The thermal degradation of POM-H extended over a broad temperature range, with complete decomposition occurring around 305 °C for both POM-H materials. In comparison, POM-C materials showed a higher initial degradation temperature, beginning slowly at temperatures of approximately 250 °C with decomposition concluding at higher temperatures, around 325 °C. Both POM-C materials underwent a more rapid main thermal decomposition after the slow initial phase compared to the faster initial decomposition and rather gradual main degradation process of the POM-H materials. This behavior indicates that the POM-H materials are more sensitive to even a small increase in temperature and are susceptible to thermal degradation in these situations during compounding or injection molding. The isothermal degradation behavior resembles the general insights that were gained by the dynamic measurements. Over a period of 60 min, both POM-C materials exhibited thermal stability at 200 °C, with no significant indications of thermal degradation. At a temperature of 220 °C, a gradual onset of thermal degradation was perceived, resulting in a total weight loss of approximately 8% after 60 min. When exposed to 240 °C, a temperature near the onset temperature determined through dynamic thermal analysis, a rapid progression of thermal degradation occurred, ultimately leading to near-complete decomposition of the POM-C materials within an hour. Notably, the higher viscosity POM-C showed a slightly improved thermal stability compared to the lower viscosity variant. In this concern, Pielichowska reported a slight decrease in thermal stability when lowering the mass-average molecular mass for neat POM-C and POM-C nanocomposites [34]. The same trend can also be observed for POM-H materials; however, they exhibit significantly lower thermal stability compared to POM-C, as previously demonstrated by the dynamic analysis. At a temperature of 220 °C, POM-H underwent an almost near-complete degradation within approximately one hour. Further increasing the temperature to 240 °C results in rapid and complete polymer degradation, with a degradation half-life of approximately 17 min. In general, the degradation rate increases with higher temperatures and is consistently faster for POM-H compared to POM-C.

#### 3.2.2. Rotational Rheometry—Time-Sweep Experiments

**Figure 5:**
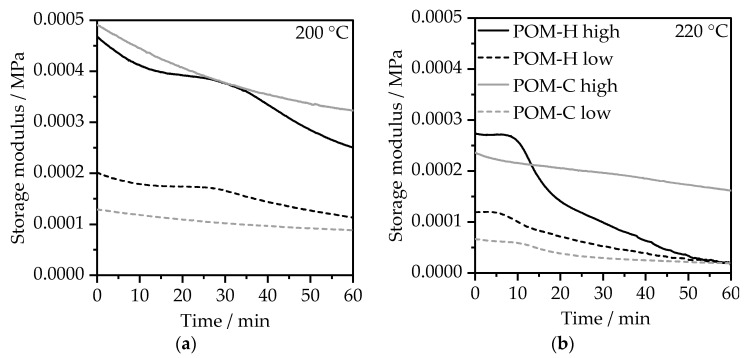
Temperature- and time-dependent change in storage modulus of the different neat POM materials in air atmosphere for the evaluation of structural changes at (**a**) 200 °C and (**b**) 220 °C, determined in a time-sweep experiment at a constant angular frequency of 10 rad/s and a constant strain of 1%.

The temperature- and time-dependent rheological behavior in air depicted by the storage modulus G′ is shown in Figure 5. The storage modulus can be used to evaluate structural changes within the polymer architecture as a result of thermal degradation, as also demonstrated by Kruse and Wagner in their observations via time-resolved mechanical spectroscopy for the degradation of Polyethyleneterephthalate [35]. At 200 °C, the storage modulus slowly decreased for both material types and both viscosity levels, which indicates the degradation of the materials. For the POM-H materials, however, the decline happened rapidly at an initial stage and in a second stage after approximately 25–30 min, which can be linked to the beginning of the thermal degradation, as also demonstrated through the isothermal TGA measurements in Figure 4b. When observing the storage modulus at an elevated temperature of 220 °C, the gradual decomposition of the POM-C materials, which was also observed in TGA, is also evident within the time-sweep experiments and can be determined by the decrease in G2 over time. The rapid decline of the storage modulus for POM-H is a clear indicator of poor thermal stability and major structural changes already after 10 min of temperature exposure. This is even more important when considering that POM-H usually requires higher processing temperatures than POM-C due to their higher melting temperature given by their chemical composition [36]. Overall, the observations in time-sweep rheometry resemble the insights obtained from TGA in Figure 4. Although a qualitative and comparative evaluation of degradation can be conducted via this method, the absolute values should be considered with care, as the material decomposes during the measurement, which results in overall inhomogeneous measuring conditions.

### 3.3. Feedstock Properties

#### 3.3.1. Filler–Matrix Interaction

**Figure 6:**
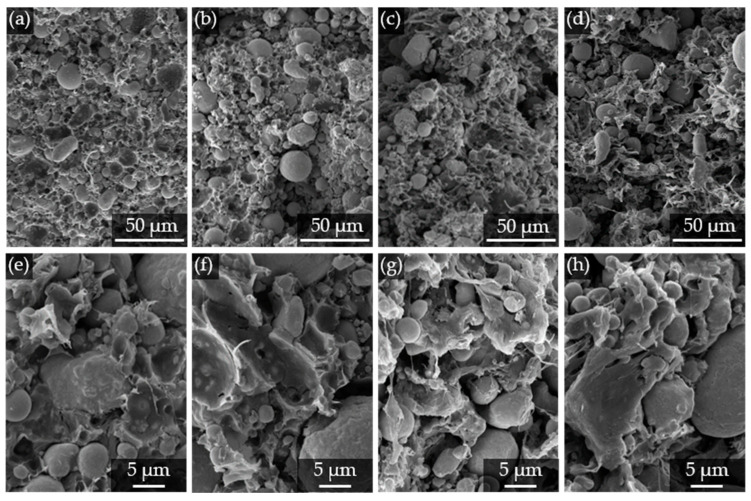
SEM images of fracture surfaces of the compounded feedstocks for (**a**,**e**) “POM-H high”; (**b**,**f**) “POM-H low”; (**c**,**g**) “POM-C high”; and (**d**,**h**) “POM-C low” at (**a**–**d**) 400× and (**e**–**h**) 2000× magnification for the evaluation of particle–matrix interactions.

The SEM images in Figure 6 show fracture surfaces of the compounded feedstock for an evaluation of the filler–matrix interactions for the different types of POMs. This assessment can be made based on the wetting behavior of the particles by the matrix. Generally, the particles are well embedded within the matrix for all materials, ensuring an even distribution within the polymer. This can be seen by the infiltration of the matrix between the particles, prohibiting the formation of agglomerates. In this regard, a uniform inter-particle spacing is desired for MIM compounds, and acts as an indicator for a homogeneous particle distribution [16]. The particle wetting behavior by the matrix, however, still offers potential for improvement, as the particle surfaces are exposed with no remaining matrix covering them. This may be achieved by further functionalizing the surface of the metal filler [37] or adding waxes [33], for instance. Furthermore, when looking into the breaking surface morphology, POM-H tends to have a more brittle mode of failure, compared to POM-C, which shows a characteristic morphology for a higher ductility, demonstrated by the fibrillation of the matrix. This behavior is especially evident within Figure 6g,h.

#### 3.3.2. Catalytic Debinding Behavior

**Figure 7:**
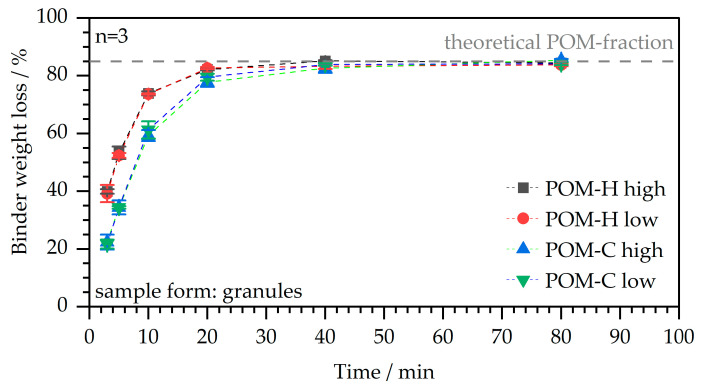
Time-dependent weight loss in the catalytic debinding process for the compounded feedstocks based on varying POM main binder components.

The debinding behavior is a crucial factor within the MIM process chain and determines the overall process time. The catalytic debinding is a comparably fast primary debinding method [21] and was evaluated for the different POM-based feedstocks for varying debinding times within Figure 7.

The curves for both material types show a rapid initial binder weight loss up to a debinding time of around 20 min, which can be attributed to the acidic attack by the nitric acid within the debinding furnace atmosphere on the polyacetal chains. Here, the homopolymers experience a faster initial degradation compared to the copolymer materials. This fact can be attributed to the higher fraction of C-O-bonds within the homopolymers, and therefore, an easier propagation of chain degradation from the chain end in the following reaction after the initial chain scission reaction [24,29]. Both material types, however, assimilate to each other and almost reach a plateau state at around 20 min debinding time with a debound fraction of around 80 wt.-%. The debinding is completed after around 40 min with a total amount of 84–85 wt.-% of binder removed. This fraction matches the amount of POM initially added within the binder fraction of the compound, and as a result, confirms a complete debinding.

#### 3.3.3. Thermal Debinding Behavior

**Figure 8:**
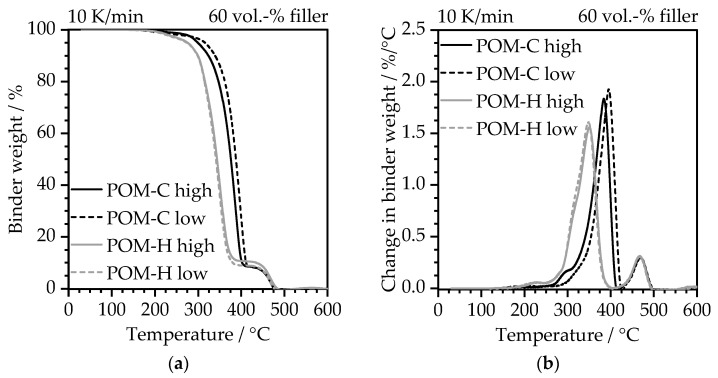
Thermal debinding behavior of feedstocks based on different POM main binder components in nitrogen at 10 K/min related to the binder weight within the feedstock: temperature dependent (**a**) binder weight and (**b**) change in binder weight.

The thermal debinding behavior in nitrogen is illustrated in Figure 8 by the weight loss of the binder fraction within the highly filled compounds prior to catalytic debinding.

All curves showcased in Figure 8a demonstrate a two-stage decomposition process, which can be clearly seen by their deviations in Figure 8b. The first major degradation step that transfers to a peak in Figure 8b can be mainly attributed to the POM component with the minor addition of stearic acid. These components correspond to approximately 88 wt.-% of the binder, as demonstrated by the decomposition residue after this stage. It has to be noted that when applying catalytic debinding prior to thermal debinding, this first decomposition stage will not be present. However, in the case of pure thermal debinding, this first major degradation step that has to be taken into account and must be passed with very low heating rates depending on the wall thickness to avoid sudden gas accumulation and formation of cracks within the part. In this context, POM-H shows an earlier decomposition onset temperature, which resembles the observations for the neat POM materials in air atmosphere from Figure 4. This behavior can be attributed to the chain chemistry of the homopolymers and the larger number of possible points of attack in the molecular chain after the initial reaction [24,29]. Furthermore, the presence of metal powders can lead to a reduced thermal stability especially for POM-H [29]. The second degradation stage is clearly separated from the first step, which begins at approximately 420 °C and ends at 500 °C. This degradation step represents the decomposition of the PE-HD backbone component. This information is crucial for the design of the thermal debinding stage after catalytic debinding. In this case, an isothermal stage before the start of the second decomposition stage at 400 °C should be introduced in order to ensure a homogeneous and gentle binder removal and avoid crack formation.

### 3.4. Processability in MIM

**Figure 9:**
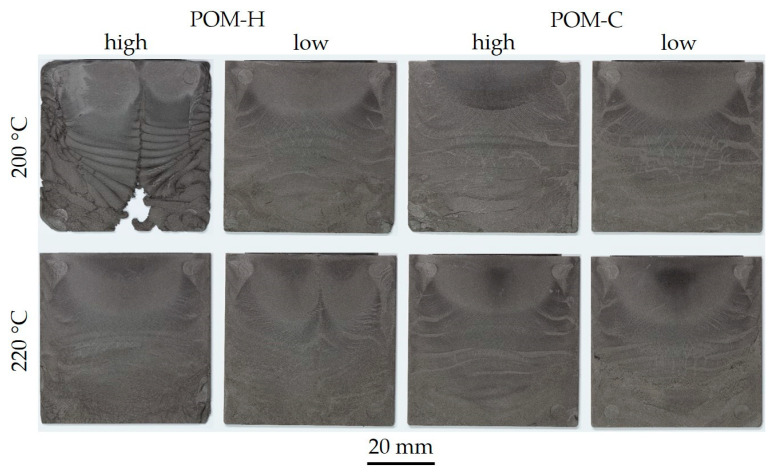
Representative green parts based on feedstocks with varying POM types manufactured at mass temperatures of 200 °C and 220 °C with the gate on the upper edge.

The processability of the compounds was evaluated within injection molding trials for two different mass temperatures. Representative samples of the corresponding injection-molded plates are shown in Figure 9.

**Figure 10:**
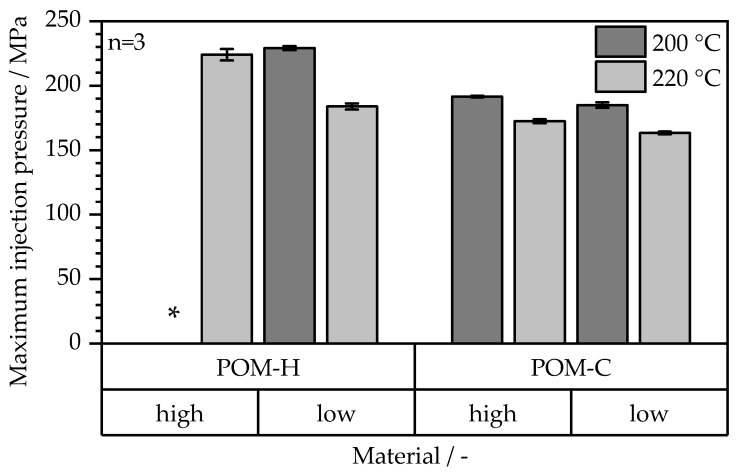
Maximum injection pressure in MIM for the feedstock materials at 200 °C and 220 °C mass temperature (*: complete form filling was not possible for the material at the given parameter combination).

The resulting maximum injection pressure was analyzed for the two temperatures for all four material combinations, as depicted by Figure 10. All manufactured compounds examined in this study were successfully processed under the given conditions. However, the form-filling capability was strongly influenced by viscosity, particularly at 200 °C. As shown in Figure 9, higher viscosity variants exhibited difficulties in achieving sufficient mold filling at 200 °C, with POM-H displaying inadequate flow characteristics under these conditions. Among the tested materials, the best results in terms of part quality and process stability were observed for the low-viscosity POM-C. However, flow patterns, indicative of particle inhomogeneity, were present in all compounds and across all investigated processing settings. These segregation effects are undesirable for MIM products and may lead to the formation of black lines [38] within the final sintered part. This may be enhanced through better particle wetting behavior and, therefore, optimized inter-particle spacing [16] and an optimization of processing parameters. The injection pressure values further underline the findings gained from the visual evaluation. Low-viscosity grades exhibited the lowest injection pressures, while POM-H required significantly higher pressure levels under equivalent processing conditions compared to POM-C. This is notable, given that initial material characterization (Figure 3) showed comparable viscosity values for the neat POM materials. These discrepancies suggest that structural modifications occurred during processing, leading to altered rheological behavior ultimately resulting in a deterioration of processability. A possible reason is the prolonged thermal exposure during compounding, which, in combination with mechanical stress from both compounding and the subsequent injection molding process, may enhance thermal degradation. POM-H appears to be more susceptible to structural changes caused by these detrimental effects compared to POM-C. Furthermore, elevated temperatures resulted in a reduction in injection pressure across all materials due to the simultaneous decrease in viscosity. However, for optimal reproducibility and process stability, low-viscosity POM-C emerged as the most favorable material choice.

## 4. Conclusions

The present work demonstrated the significance of the choice of the POM type within MIM feedstocks destined for catalytic debinding regarding their processing behavior. A careful binder choice is crucial, to ensure stable processing conditions over time, due to the tendency for degradation of POM. Especially when employing thermally sensitive POM-H materials as the main binder, processing in injection molding can be strongly affected due to thermal altering of the material. In this regard, POM-H materials are prone to thermal degradation even at short exposition times. As a result, the use of POM-H leads to lower processing stability and higher resulting injection pressures compared to equivalent POM-C types. The chemical constitution of POM-H, however, also results in an initially faster progress in catalytic debinding, which assimilates to the POM-C for longer debinding times offering a full removal of the POM component. Although the effect within this study was only small, this generally may offer potential for shorter debinding times in bigger or thick-walled parts and should be further studied in future investigations. Within the study, all the materials showed satisfactory processing behavior in terms of form filling, thermal, and catalytic debinding, proving their general suitability for the MIM process. The injection pressures and flow patterns, however, could be optimized by the employment of low viscosity materials and higher processing temperatures. In this regard, the low viscosity POM-C is favorable for the MIM process. Further insight may be gained in this context by systematically varying the molecular weight distributions and stabilization of the POM types, which will be addressed in future studies. In addition, future work within this field will focus on the addition of processing aids to optimize processing characteristics in terms of rheology and to improve powder–binder adhesion to avoid their separation. A targeted variation of geometrical boundary conditions and processing parameters could also help in understanding the formation of a non-uniform particle distribution within the part and should also be the focus of future investigations.

## Figures and Tables

**Table 1:** Injection molding parameters for the production of plate specimens.

Parameter/Unit	Value
Mold temperature/°C	100
Mass temperature/°C	200/220
Injection speed/mm·s^−1^	20
Holding pressure/bar	900
Time of holding pressure/s	10
Residual cooling time/s	30

## Data Availability

The data presented in this study are available upon request from the corresponding author.
